# Growth differentiation factor–15 predicts the prognoses of patients with acute coronary syndrome: a meta-analysis

**DOI:** 10.1186/s12872-016-0250-2

**Published:** 2016-05-06

**Authors:** Shangshi Zhang, Dongjun Dai, Xian Wang, Hongyan Zhu, Hongchuan Jin, Ruochi Zhao, Liting Jiang, Qi Lu, Fengying Yi, Xiangxiang Wan, Hanbin Cui

**Affiliations:** Department of Cardiovascular, Ningbo First Hospital, Ningbo, 315010 China; Department of Cardiovascular, Shangrao People ’s Hospital, Shangrao, 334000 China; Department of Medical Oncology, Institute of Clinical Science, Sir Run Run Shaw Hospital, Medical School of Zhejiang University, Hangzhou, 310029 China

## Abstract

**Background:**

Recent studies have shown Growth differentiation factor–15 (GDF-15) that is a member of the transforming growth factor β (TGF-β) superfamily might be a potential predictive cytokine for the prognosis of Acute coronary syndrome (ACS). However, there are discrepancies in these studies.

**Methods:**

Publication searches of the PubMed/Medline and EMBASE databases were performed without any time or ethnicity restrictions. The inclusion and exclusion criteria, when clear, were addressed. Random effects models were used for all analyses. Publication bias was tested using funnel plots and the Egger test.

**Results:**

We identified eight eligible studies that provided mortality data. Five of these studies provided recurrent myocardial infarction (MI) data. The maximal duration of follow-up ranged from 6 months to 6 years. A significant association was found between the patients with the highest and lowest GDF-15 levels (overall analyses) in terms of mortality (*p* < 0.00001; RR = 6.08; 95 % CI = 4.79–7.71) and recurrent MI (*p* < 0.00001; RR = 1.76; 95 % CI = 1.49–2.07). We also found significant associations between the subgroup analyses stratified by ACS types, cutoff points and follow-up durations (*p* < 0.001). The combined hazard ratio was high for GDF-15 to ACS (HR = 1.656, 95 % CI = 1.467–1.871).

**Conclusion:**

High plasma GDF-15 levels are associated with an increased risk of mortality and recurrent MI in patients with ACS.

**Electronic supplementary material:**

The online version of this article (doi:10.1186/s12872-016-0250-2) contains supplementary material, which is available to authorized users.

## Background

Acute coronary syndrome (ACS), including ST-segment elevation myocardial infarction (STEMI), non-ST-segment elevation myocardial infarction (NSTEMI) and unstable angina pectoris (UAP), result from the rupture or erosion of vulnerable atherosclerotic plaque [1], and death and recurrent myocardial infarction (MI) can occur at any time after the first attack episode. Currently, even with medication and coronary intervention, the prognosis for ACS patients is still not good [2]. Therefore, the early evaluation of ACS and its timely treatment are particularly important.

Growth differentiation factor–15 (GDF-15) is a member of the transforming growth factor β (TGF-β) superfamily. GDF-15 primarily regulates multiple cellular functions, as well as the biological processes underlying the growth of multiple organs and the differentiation and renovation of tissues [3]. Researchers have verified that GDF-15 exerts a suppressive function in the progress, invasiveness, and metastasis of tumors by facilitating the apoptosis of tumor cells [4]. In addition, GDF-15 is also related to cardiovascular diseases, such as heart failure [5], cardiac hypertrophy [6], and coronary heart disease (CHD) [7]. Recent studies have shown that GDF-15 might be a potential predictive cytokine for the prognosis of ACS and, therefore, might assist physicians in evaluating the prognoses of ACS patients [8]. Studies examining the association between GDF-15 and ACS prognosis have included research on patient mortality [7, 9–15] and recurrent MI [9–12, 15]. However, there have been discrepancies in these studies, including the target participants, follow-up period, and patient ethnicity. Hence, this meta-analysis was performed to investigate the role of GDF-15 in predicting the prognosis of ACS patients.

## Methods

### Inclusion criteria

We performed a meta-analysis to determine whether GDF-15 could predict the prognoses of ACS patients. Studies that had a follow-up duration of least 6 months and that examined the correlation between plasma GDF-15 levels and the prognoses of ACS patients were collected. The quality of each included study has been tested under the Meta-analysis of Observational Studies in Epidemiology (MOOSE) [16]. The MOOSE systemic assessments included the following criteria: 1) a clear explanation of the inclusion and exclusion criteria, 2) the attrition rate of the samples during the follow-up period, 3) a clear explanation of the outcomes and outcome assessments, 4) a sufficient follow-up period, 5) appropriate statistical analyses, and 6) identification of the important confounding and prognostic factors. All items had the following answer options: yes/no/unclear information to answer the question. When a criterion was fulfilled, a score of 1 was given; a score of 0 was given if a criterion was unclear, and −1 was given if a criterion was not achieved. The MOOSE assessment of each study was discussed and carefully evaluated by all authors.

### Study selection

The study selection was based on an online search of the PubMed/MEDLINE and EMBASE databases, without any language restrictions; studies published before November 14, 2014 were included. The following medical subject headings were used for the publication search: “ST-segment elevation myocardial infarction, STEMI, Non-ST-segment elevation myocardial infarction, NSTEMI, Unstable angina pectoris, UAP, Acute coronary syndrome, ACS, growth differentiation factor-15, GDF-15, Placental growth factor, PGF, macrophage inhibitory cytokine-1, MIC-1”. The terms were then combined using Boolean the operators “OR, AND”. We extracted the data from individual studies that included the first author’s name, year of publication, ethnicity, participants, outcome data (mortality and recurrent MI), and maximal follow-up years, as well as methods that stratified the GDP-15 levels. The extraction was performed by two independent reviewers (SZ and DD).

### Statistical analysis

Review Manager 5.3 and STATA 11.0 statistical software were used for the meta-analysis [17]. Statistical heterogeneity was tested using Cochran’s Q statistic and I^2^ tests [18]. The random effects model was used for all meta-analyses. The calculated GDF-15 concentration data were extracted from the initial follow-up, and *p* < 0.05 was considered to be statistically significant.

## Results

### Study selection

**Fig. 1:**
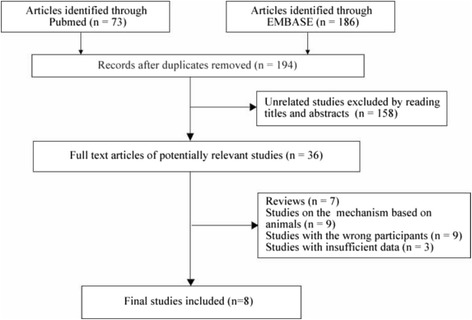
Flowchart of selection process in the meta-analysis

We retrieved 73 studies from PubMed/MEDLINE and 186 studies from EMBASE (Fig. 1). After removing duplicates, 158 of 194 studies were excluded based on title and abstract reviews. In the remaining 36 studies, there were seven reviews, nine studies based on animals, nine studies with different participants, and three studies with inadequate data. Finally, eight articles were included in our meta-analysis, including 4 NSTEMI studies, 2 STEMI studies and 2 other studies that did not clearly provide the types of ACS. No unstable angina pectoris data were found.

Characteristics of the individual studies are shown in Table 1. Eight studies, including 8903 participants, investigated the association between GDF-15 and mortality. Five of these studies, including 7193 participants, investigated the association between GDF-15 and recurrent MI. The maximal follow-up duration ranged from 6 months to 6 years.

**Table 1:** The characteristics of the studies included in the meta-analysis

Author/year/ethnicity	Participants	Outcomes	Follow-up^a^	Comparisons (ng/L)	RR [95 %CI]^b^	MOOSE
Kempf T/2009/Europeans [7]	ACS (*n* = 877)	M	6	<1200, 1200–1800, >1800	8.5 [3.81, 18.99]	6
Bonaca MP/2011/Europeans [9]	ACS (*n* = 3501)	M, R	2	<1362	4.99 [2.82, 8.83]	5
				≥1362		
Wollert KC/2007(1)/Europeans [10]	NSTEMI (*n* = 2079)	M, R	2	<1200, 1200–1800, >1800	6.11 [3.35, 11.16]	6
Damman P/2014/Europeans [11]	NSTEMI (*n* = 1151)	M, R	5	<1200, 1200–1800, >1800	5.54 [3.18, 9.64]	6
Wollert KC/2007(2)/Europeans [12]	NSTEMI (*n* = 2081)	M, R	1	<1200, 1200–1800, >1800	9.12 [4.93, 16.85]	4
Eggers KM/2010/Europeans [13]	NSTEMI (*n* = 950)	M	5	<1200, 1200–1800, >1800	4.3 [2.44, 7.58]	6
Kempf T/2007/Europeans [14]	STEMI (*n* = 741)	M	1	<1200, 1200–1800, >1800	6.66 [2.43, 18.23]	6
Eitel I/2011/Europeans [15]	STEMI (*n* = 238)	M, R	0.5	<1319	19 [2.58, 139.66]	4
				≥1319		

*SAP* stable angina pectoris, *ACS* acute coronary syndrome, *NSTEMI* non-ST-elevation myocardial infarction, *STEMI* ST-elevation myocardial infarction, *M* mortality, *R* recurrent MI, ^a^the maximum follow up year, ^b^the calculated risk radio based on the mortality, *MOOSE* meta-analysis of observational studies in epidemiology

### Association between GDF-15 and the ACS prognosis

In addition to comparing the prognoses of the ACS patients with the highest and lowest GDF-15 levels (overall analyses), we also performed subgroup analyses based on the ACS types and a stratification of the GDF-15 values: <1200 ng/L, 1200–1800 ng/L, and >1800 ng/L. Moreover, based on the included studies, we stratified the follow-up duration into “>1 years” and “≤1 years”. Patient mortality and recurrent MI data were analyzed (Table 2, Additional file 1: Figure S1 and Additional file 2: Figure S2).

**Table 2:** The association between GDF-15 concentration and ACS prognosis

	Comparison	Participants	RR [95 %CI]	*p* value	I^2^
Mortality					
ACS type	Overall	8903	6.08 [4.79, 7.71]	<0.00001	0 %
	ACS	4123	6.03 [3.66, 9.93]	<0.00001	11 %
	NSTEMI	4038	5.94 [4.38, 8.05]	<0.00001	8 %
	STEMI	742	8.24 [3.35, 20.25]	<0.00001	0 %
Cutoff Point	<1200 VS 1200–1800	5551	2.23 [1.66, 2.99]	<0.00001	0 %
	1200–1800 VS >1800	4985	2.76 [2.28, 3.34]	<0.00001	0 %
	<1200 VS >1800	5164	6.22 [4.77, 8.10]	<0.00001	0 %
Follow up duration	>1 years	6744	5.47 [4.18, 7.16]	<0.00001	0 %
	≤1 years	2159	8.83 [5.32, 14.66]	<0.00001	0 %
Recurrence of MI					
ACS type	Overall	7193	1.76 [1.49, 2.07]	<0.00001	5 %
	ACS	3501	2.02 [1.56, 2.61]	<0.00001	NA
	NSTEMI	3454	1.66 [1.35, 2.03]	<0.00001	0 %
	STEMI	238	1.00 [0.39, 2.58]	1.00	NA
Cutoff Point	<1200 VS 1200–1800	3766	1.10 [0.75, 1.59]	0.64	65 %
	1200–1800 VS >1800	3402	1.49 [1.17, 1.89]	0.0010	27 %
	<1200 VS >1800	3454	1.66 [1.35, 2.03]	<0.00001	0 %
Follow up duration	>1 years	5538	1.84 [1.54, 2.19]	<0.00001	5 %
	≤1 years	1655	1.42 [0.96, 2.08]	0.08	0 %

*NA* not applicable, the units of GDF-15 concentration is ng/L

For the mortality analyses, a significant association was found in the overall analyses (*p* < 0.00001, RR = 6.08, 95 % CI = 4.79–7.71) and subgroup analyses of ACS (*p* < 0.00001, RR = 6.03, 95 % CI =3.66–9.93), NSTEMI (*P* < 0.00001, RR = 5.94, 95 % CI = 4.38–8.05) and STEMI (*p* < 0.00001, RR = 8.24, 95 % CI = 3.35–20.25). Positive results were also revealed in the subgroup analyses stratified by GDF-15 Cutoff Point (<1200 ng/L vs. 1200–1800 ng/L:*p* < 0.00001, RR = 2.23, 95 % CI = 1.66–2.99; 1200–1800 ng/L vs. > 1800 ng/L:*p* < 0.00001, RR = 2.76, 95 % CI = 2.28–3.34; and >1800 ng/L vs. < 1200 ng/L:*p* < 0.00001 ng/L, RR = 6.22, 95 % CI = 4.77–8.10). As for subgroup analyses stratified by follow-up duration, positive results were also obtained for the ≤1 year (*p* < 0.00001, RR =8.83, 95 % CI = 5.32–14.66) and >1 year subgroups (*p* < 0.00001, RR = 5.47, 95 % CI = 4.18–7.16).

For the recurrent MI analyses, a significant association was observed in the overall analyses (*p* < 0.00001, RR = 1.76, 95 % CI = 1.49–2.07) and in the subgroup analyses of NSTEMI (*p* < 0.00001, RR = 1.66, 95 % CI = 1.35–2.03) and ACS (*p* < 0.00001, RR = 2.02, 95 % CI = 1.56–2.61). A significant association was also found when comparing the following GDF-15 cutoff points: 1200–1800 ng/L vs. >1800 ng/L (*p* < 0.00001, RR = 1.49, 95 % CI = 1.17–1.89) and >1800 ng/L vs. <1200 ng/L (*p* < 0.00001, RR = 1.66, 95 % CI = 1.35–2.03). As for the subgroup analyses stratified by the follow-up durations, a positive result was found only in the >1 year follow-up duration (*p* < 0.00001, RR =1.84, 95 % CI =1.54–2.19); we did not find a significant result for follow-up periods ≤1 year (*P* = 0.08, RR = 1.42, 95 % CI = 0.96–2.08).

### Heterogeneity of the current study

High heterogeneity was found in the comparison of the recurrent MI between the GDF-15 levels of <1200 ng/L and 1200 to 1800 ng/L (I^2^ = 65 %). Heterogeneity was low for all the above mentioned analyses (I^2^ <27 %).

### The combined prognostic data of hazard ratio

**Fig. 2:**
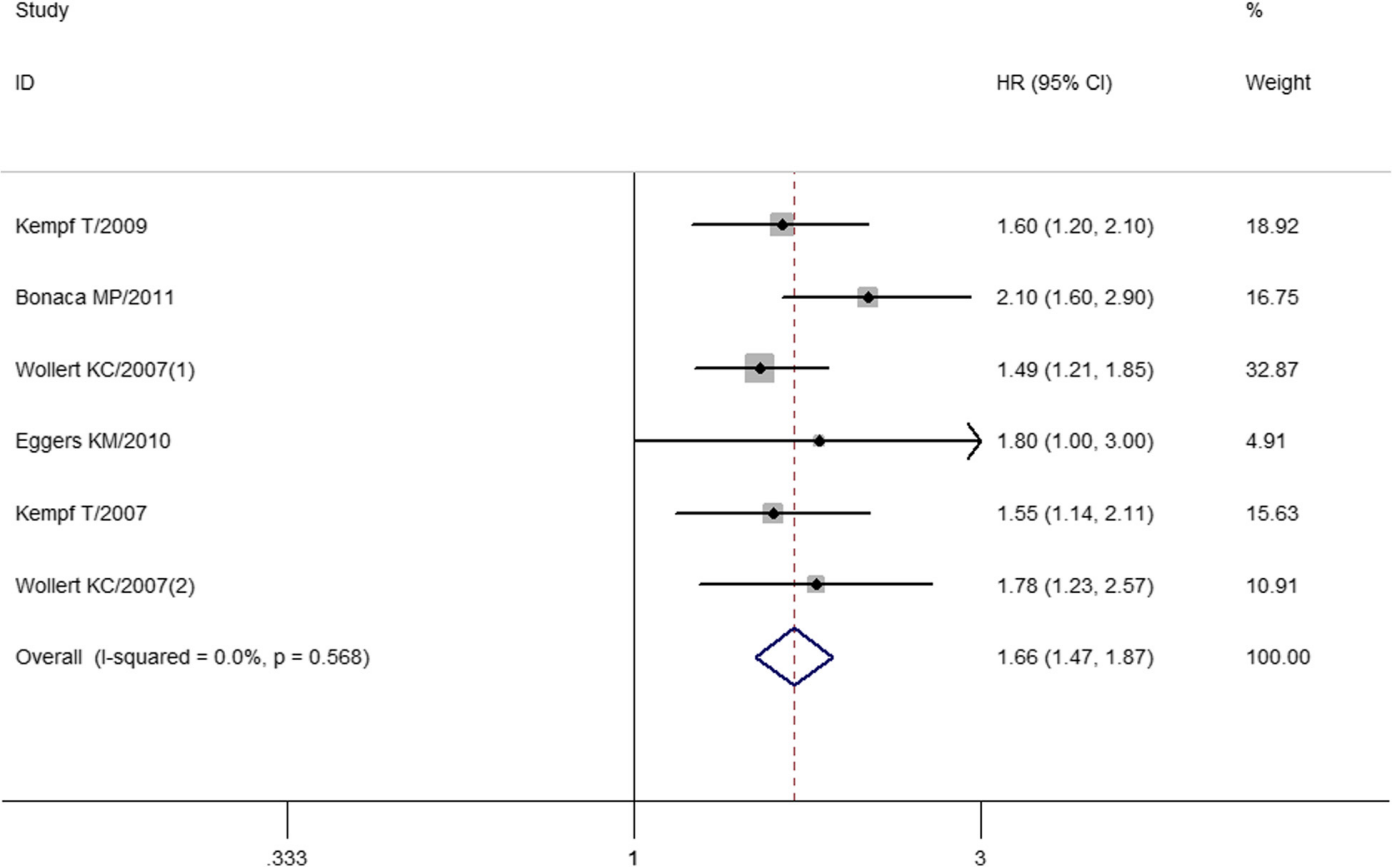
Funnel plots of GDF-15 with mortality and recurrent MI

Hazard ratio (HR) was obtained in seven studies [7, 9, 10, 12–15], which was on the association of the GDF-15 and mortality or recurrent of MI. However, after sensitive analysis, we excluded a study [15] that could lead to high heterogeneity to the overall meta-analysis. Our results showed a significant high HR (HR = 1.656, 95 % CI = 1.467–1.871, I^2^ = 0 %) for GDF-15 to the ACS (Fig. 2).

## Discussion

Our meta-analysis identified eight studies, including 8903 participants, and attempted to evaluate the predictive value of plasma GDF-15 levels in the prognosis of ACS patients by calculating the risk of mortality and recurrent MI after the ACS events. We found that higher GDF-15 levels indicated a higher risk for ACS mortality and recurrent MI. This association also remained when the GDF-15 levels were stratified by the cutoff point, which might be valuable in terms of the risk stratification of ACS prognoses because it has been previously reported that 1800 ng/L GDF-15 levels may be an ideal cutoff value for several disease states [19]. Moreover, we also found significant associations between GDF-15 levels and subsequent ACS events. Furthermore, we found high HR for GDF-15 to ACS. These findings might assist with monitoring the prognoses of ACS patients.

GDF-15 has been described as a promising cardio-protective agent [20]. Previous studies have reported that higher expression levels of GDF-15 could predict deteriorating conditions for heart disease patients, particularly ACS patients, independent of troponin or BNP levels [7, 9–15]. It has also been reported that GDF-15 plays a protective role in heart disease, which may be attributed to the anti-apoptotic, anti-inflammatory, or anti-hypertrophic effects demonstrated in animal models [21, 22]. Moreover, GDF-15 might induce angiogenesis, which plays an essential role in the recovery of damaged myocardium [23]. A prolonged increase in GDF-15 levels after episodes of ischemia and reperfusion may be associated with increased levels of oxidative stress, inflammation, and infarct healing, and the increase of GDF-15 requires for enhanced oxidative metabolism and tissue repair [9]. Accordingly, one feasible explanation for the predictive role of GDF-15 is that higher levels of GDF-15 indicate a greater extent of myocardium damage and the risk of adverse remodeling. GDF-15 is also strongly induced in other cardiovascular conditions, such as heart failure [21], Takotsubo cardiomyopathy [24], and atherosclerosis [25].

**Fig. 3:**
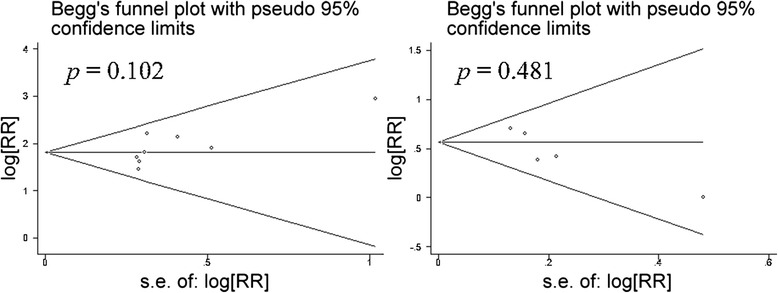
The forest plots of combined hazard rate of GDF-15 with mortality or recurrent of MI

In this meta-analysis, clear inclusion and exclusion criteria were required for study inclusion. The quality of the current study was well assessed using the MOOSE system. There was no publication bias, according to the funnel plots (Fig. 3). Heterogeneity was high among the “<1200 ng/L vs. 1200–1800 ng/L” subgroup of recurrent MI patients. The following sensitive test showed that this heterogeneity disappeared (I^2^ = 0 %) when we removed an included study from the Netherlands [11], thus indicating that the heterogeneity might have resulted from ethnicity differences, as the remaining two studies included samples from Sweden. The other comparisons showed low or no heterogeneity (I^2^ < = 27 %), suggesting that our study results were reliable.

There are ethnic differences in GDF-15 levels. In Europeans, the median GDF-15 concentration ranged from 1244 to 1635 ng/L, whereas it was much lower in Asians (650 ng/L) [26]. However, the Asian study cohort was limited in number, and future well-designed studies with different ethnicities are warranted.

### Limitations

Although individual patient data from eight eligible studies were included in our meta-analysis, this study had some limitations. First, the definitions of all-cause mortality and cardiovascular mortality were not confirmed. Among the studies included, only two studies provided detailed information [7, 9]. For the recurrent MI patients without causes of death, we only performed subgroup analyses for “recurrent MI”. Second, although the revealed heterogeneity was low for most of the comparisons, GDF-15 levels might be influenced by many factors, including age, male gender, current smoking, symptomatic heart failure, reduced kidney function, and diabetes mellitus, among others [27]. Third, the ACS patients were treated with different treatment strategies, which might alter the GDF-15 levels. Fourth, the obtained blood samples were extracted at different times, therefore the GDF-15 levels may change by different time after ACS events, however, evidence found that GDF-15 levels showed small alterations several months after ACS events [28], hence, we could not give a judgment if the different time of the tests of the GDF-15 would influence our findings. Fifth, the follow-up durations differed in each study. In addition, one study [13] recorded the initial mortality rate 6 months after the ACS occurred, which might slightly alter our study results. Sixthly, the HR of different studies were obtained at different time after the ACS happened, which may slightly influence the results, however, the combined data showed no heterogeneity (I^2^ = 0 %).

## Conclusion

The present study suggests that GDF-15 is a strong predictor of mortality and recurrent MI in ACS prognosis. Thus, it has the potential to become a clinically useful novel biomarker that can provide independent prognostic information and also help to direct optimal treatment strategies. Current ongoing clinical trials are needed to further clarify the benefits of GDF-15 in predicting the prognoses of patients with acute coronary syndrome.

### Ethics

This study is not involved in any ethical issues.

## Additional files

Additional file 1: Figure S1. Forest plots of GDF-15 with mortality. (TIF 28 kb) Additional file 2: Figure S2. Forest plots of GDF-15 with recurrent of MI. (TIF 25 kb)

## Abbreviations

GDF-15: growth differentiation factor–15
ACSm: acute coronary artery syndrome
MI: myocardial infarction
STEMI: ST-segment elevation myocardial infarction
NSTEMI: non-ST-segment elevation myocardial infarction
UAP: unstable angina pectoris
CHD: coronary heart disease
MOOSE: meta-analysis of observational studies in epidemiology

## References

[1] Vedanthan R, Seligman B, Fuster V (2014). Global perspective on acute coronary syndrome: a burden on the young and poor. Circ Res.

[2] Fath-Ordoubadi F, Barac Y, Abergel E, Danzi GB, Kerner A, Nikolsky E, Halabi M, Mamas M, El-Omar M, Fraser D (2012). Gender impact on prognosis of acute coronary syndrome patients treated with drug-eluting stents. Am J Cardiol.

[3] Ago T, Sadoshima J (2006). GDF15, a cardioprotective TGF-beta superfamily protein. Circ Res.

[4] Mimeault M, Batra SK (2010). Divergent molecular mechanisms underlying the pleiotropic functions of macrophage inhibitory cytokine-1 in cancer. J Cell Physiol.

[5] Anand IS, Kempf T, Rector TS, Tapken H, Allhoff T, Jantzen F, Kuskowski M, Cohn JN, Drexler H, Wollert KC (2010). Serial measurement of growth-differentiation factor-15 in heart failure: relation to disease severity and prognosis in the Valsartan Heart Failure Trial. Circulation.

[6] Dominguez-Rodriguez A, Abreu-Gonzalez P, Avanzas P (2011). Relation of growth-differentiation factor 15 to left ventricular remodeling in ST-segment elevation myocardial infarction. Am J Cardiol.

[7] Kempf T, Sinning JM, Quint A, Bickel C, Sinning C, Wild PS, Schnabel R, Lubos E, Rupprecht HJ, Munzel T (2009). Growth-differentiation factor-15 for risk stratification in patients with stable and unstable coronary heart disease: results from the AtheroGene study. Circ Cardiovasc Genet.

[8] Lange RA (2013). Can you predict what happens when EuroSCORE weds biomarker?. J Am Coll Cardiol.

[9] Bonaca MP, Morrow DA, Braunwald E, Cannon CP, Jiang S, Breher S, Sabatine MS, Kempf T, Wallentin L, Wollert KC (2011). Growth differentiation factor-15 and risk of recurrent events in patients stabilized after acute coronary syndrome: observations from PROVE IT-TIMI 22. Arterioscler Thromb Vasc Biol.

[10] Wollert KC, Kempf T, Lagerqvist B, Lindahl B, Olofsson S, Allhoff T, Peter T, Siegbahn A, Venge P, Drexler H (2007). Growth differentiation factor 15 for risk stratification and selection of an invasive treatment strategy in non ST-elevation acute coronary syndrome. Circulation.

[11] Damman P, Kempf T, Windhausen F, van Straalen JP, Guba-Quint A, Fischer J, Tijssen JG, Wollert KC, de Winter RJ, Hirsch A (2014). Growth-differentiation factor 15 for long-term prognostication in patients with non-ST-elevation acute coronary syndrome: an Invasive versus Conservative Treatment in Unstable coronary Syndromes (ICTUS) substudy. Int J Cardiol.

[12] Wollert KC, Kempf T, Peter T, Olofsson S, James S, Johnston N, Lindahl B, Horn-Wichmann R, Brabant G, Simoons ML (2007). Prognostic value of growth-differentiation factor-15 in patients with non-ST-elevation acute coronary syndrome. Circulation.

[13] Eggers KM, Kempf T, Lagerqvist B, Lindahl B, Olofsson S, Jantzen F, Peter T, Allhoff T, Siegbahn A, Venge P (2010). Growth-differentiation factor-15 for long-term risk prediction in patients stabilized after an episode of non-ST-segment-elevation acute coronary syndrome. Circ Cardiovasc Genet.

[14] Kempf T, Bjorklund E, Olofsson S, Lindahl B, Allhoff T, Peter T, Tongers J, Wollert KC, Wallentin L (2007). Growth-differentiation factor-15 improves risk stratification in ST-segment elevation myocardial infarction. Eur Heart J.

[15] Eitel I, Blase P, Adams V, Hildebrand L, Desch S, Schuler G, Thiele H (2011). Growth-differentiation factor 15 as predictor of mortality in acute reperfused ST-elevation myocardial infarction: insights from cardiovascular magnetic resonance. Heart.

[16] Stroup DF, Berlin JA, Morton SC, Olkin I, Williamson GD, Rennie D, Moher D, Becker BJ, Sipe TA, Thacker SB (2000). Meta-analysis of observational studies in epidemiology: a proposal for reporting. Meta-analysis Of Observational Studies in Epidemiology (MOOSE) group. JAMA.

[17] Kawalec P, Mikrut A, Wisniewska N, Pilc A (2013). The effectiveness of tofacitinib, a novel Janus kinase inhibitor, in the treatment of rheumatoid arthritis: a systematic review and meta-analysis. Clin Rheumatol.

[18] Coory MD (2010). Comment on: Heterogeneity in meta-analysis should be expected and appropriately quantified. Int J Epidemiol.

[19] Rohatgi A, de Lemos JA (2009). The report card on growth differentiation factor 15: consistent marks but not yet ready for promotion. Circ Cardiovasc Genet.

[20] Wollert KC (2007). Growth-differentiation factor-15 in cardiovascular disease: from bench to bedside, and back. Basic Res Cardiol.

[21] Kempf T, Zarbock A, Widera C, Butz S, Stadtmann A, Rossaint J, Bolomini-Vittori M, Korf-Klingebiel M, Napp LC, Hansen B (2011). GDF-15 is an inhibitor of leukocyte integrin activation required for survival after myocardial infarction in mice. Nat Med.

[22] Kempf T, Eden M, Strelau J, Naguib M, Willenbockel C, Tongers J, Heineke J, Kotlarz D, Xu J, Molkentin JD (2006). The transforming growth factor-beta superfamily member growth-differentiation factor-15 protects the heart from ischemia/reperfusion injury. Circ Res.

[23] Yamashita H, Shimizu A, Kato M, Nishitoh H, Ichijo H, Hanyu A, Morita I, Kimura M, Makishima F, Miyazono K (1997). Growth/differentiation factor-5 induces angiogenesis in vivo. Exp Cell Res.

[24] Stiermaier T, Adams V, Just M, Blazek S, Desch S, Schuler G, Thiele H, Eitel I (2014). Growth differentiation factor-15 in Takotsubo cardiomyopathy: diagnostic and prognostic value. Int J Cardiol.

[25] Brown DA, Breit SN, Buring J, Fairlie WD, Bauskin AR, Liu T, Ridker PM (2002). Concentration in plasma of macrophage inhibitory cytokine-1 and risk of cardiovascular events in women: a nested case-control study. Lancet.

[26] Lin JF, Wu S, Hsu SY, Yeh KH, Chou HH, Cheng ST, Wu TY, Hsu WT, Yang CC, Ko YL (2014). Growth-differentiation factor-15 and major cardiac events. Am J Med Sci.

[27] Pati PK, George PV, Jose JV (2013). Giant pulmonary artery aneurysm with dissection in a case of Marfan syndrome. J Am Coll Cardiol.

[28] Frankenstein L, Remppis A, Frankenstein J, Hess G, Zdunek D, Gut S, Slottje K, Katus HA, Zugck C (2009). Reference change values and determinants of variability of NT-proANP and GDF15 in stable chronic heart failure. Basic Res Cardiol.

